# Curcumin Conjugated with PLGA Potentiates Sustainability, Anti-Proliferative Activity and Apoptosis in Human Colon Carcinoma Cells

**DOI:** 10.1371/journal.pone.0117526

**Published:** 2015-02-18

**Authors:** Bhargav N. Waghela, Anupama Sharma, Suhashini Dhumale, Shashibahl M. Pandey, Chandramani Pathak

**Affiliations:** 1 Department of Cell Biology, Indian Institute of Advanced Research, Gandhinagar, Gujarat, India; 2 Department of Zoology, Smt. C.H.M. College, Ulhasnagar, Maharashtra, India; Rajiv Gandhi Centre for Biotechnology, INDIA

## Abstract

Curcumin, an ingredient of turmeric, exhibits a variety of biological activities such as anti-inflammatory, anti-atherosclerotic, anti-proliferative, anti-oxidant, anti-cancer and anti-metastatic. It is a highly pleiotropic molecule that inhibits cell proliferation and induces apoptosis in cancer cells. Despite its imperative biological activities, chemical instability, photo-instability and poor bioavailability limits its utilization as an effective therapeutic agent. Therefore, enhancing the bioavailability of curcumin may improve its therapeutic index for clinical setting. In the present study, we have conjugated curcumin with a biodegradable polymer Poly (D, L-lactic-co-glycolic acid) and evaluated its apoptotic potential in human colon carcinoma cells (HCT 116). The results show that curcumin-PLGA conjugate efficiently inhibits cell proliferation and cell survival in human colon carcinoma cells as compared to native curcumin. Additionally, curcumin conjugated with PLGA shows improved cellular uptake and exhibits controlled release at physiological pH as compared to native curcumin. The curcumin-PLGA conjugate efficiently activates the cascade of caspases and promotes intrinsic apoptotic signaling. Thus, the results suggest that conjugation potentiates the sustainability, anti-proliferative and apoptotic activity of curcumin. This approach could be a promising strategy to improve the therapeutic index of cancer therapy.

## Introduction

Colon cancer is the fourth leading cause of death and third most common cancer worldwide [1]. Progression of colon cancer is very aggressive and has a very low survival rate due to lack of effective therapy. At present, conventional chemotherapy, surgery and radiation therapy suffer from major obstacles due to reoccurrence, resistance and adverse side effects. Broadly, various natural polyphenolic compounds are known to have an anti-cancerous properties but poor bioavailability limits their clinical setting [2].

Curcumin (1,7-Bis (4-hydroxy-3-methoxyphenyl)-1,6-heptadiene-3,5-dione) is a natural polyphenolic compound derived from the rhizome of the plant *Curcuma longa*, which is known to have a broad spectrum of biological activities including anti-oxidant, anti-inflammatory, anti-proliferative, anti-angiogenic, anti-microbial, anti-parasitic and immunomodulatory [3–5]. Curcumin has been shown to have pleiotropic effects by modulating many cellular targets such as transcription factor nuclear factor kappa B (NF-κB), transcription factor activator protein-1 (AP-1), c-Myc, c-Jun N-terminal kinase, protein kinase C, protein tyrosine kinases, protein serine/threonine kinases and many other proteins [6–8]. It also inhibits secretion of interleukins and cytokines as well as an expression of human epidermal growth factor receptor (EGFR) [9]. Thus, curcumin has remarkable ability to modulate a wide variety of signaling pathways associated with tumorigenesis, cell proliferation, invasion, apoptosis and cell cycle regulation [10, 11]. It has an ability to overcome resistance of anticancer drugs and further potentiate their anti-cancer activity [12, 13]. The recent report of phase I clinical trials suggest that curcumin is a promising chemopreventive agent for pre-malignant cells and non-toxic to humans for up to 8 g/day when taken up to 3 months [14, 15]. Phase II clinical trials of curcumin also demonstrate that curcumin is well tolerated when administered orally and exhibits the biological activity despite its limited absorption against pancreatic cancer [16, 17].

Despite its valuable potential, pharmacological efficacy is limited primarily due to its water insolubility, chemical instability, photo-instability, rapid metabolism, limited tissue distribution and poor absorption that ultimately lead to poor bioavailability of this compound [18, 19]. Therefore, several approaches have been applied to improve bioavailability and therapeutic potential include modification of the parental structure, synthesis of stable derivatives and the formulation of nanoparticles [20–22]. In the present study, we have synthesized the stable derivative of curcumin by conjugating it with poly (D, L-lactic-co-glycolic acid) (PLGA) polymer. It is a biocompatible, biodegradable, non-toxic and non-immunogenic polymer that has excellent properties for drug delivery [20, 23]. The conjugation improves sustainability and ultimately biological properties of curcumin. Thus, we have investigated the sustainability and cellular uptake of curcumin-PLGA conjugate. Further, we examined its efficiency in inhibition of cell proliferation and molecular mechanism for induction of apoptotic cell death signaling in human colon carcinoma cells.

## Materials and Methods

### Chemicals and Reagents

All chemicals of molecular biology grade were purchased commercially. Curcumin, Poly (D,L-lactic-co-glycolic acid) (PLGA), Dicyclohexylcarbodiimide (DCC), 4-(Dimethylamino) pyridine (DMAP), cell culture grade Dimethyl sulfoxide (DMSO), Poly L-lysine, 4',6-diamidino-2-phenylindole (DAPI), (3-(4,5-dimethylthiazol-2-yl)-2,5-diphenyltetrazolium bromide (MTT), Trypan Blue, 2’,7’-Dichlorofluorescein diacetate (H_2_DCFDA), N-acetyl cysteine (NAC), Bicinchoninic acid (BCA) protein estimation kit and anti-mouse HRP conjugated secondary antibody were purchased from Sigma-Aldrich (St. Louis, MO, USA). The complete, EDTA-free protease inhibitor cocktail (PIC) was purchased from Roche (Penzberg, Germany). Annexin V-FITC/Propidium Iodide (PI) apoptosis detection kit and 5,5',6,6'-Tetrachloro-1,1',3,3'-tetraethylbenzimidazolylcarbocyanine Iodide (JC-1) dye were purchased from BioVision (California, US). DMEM, RPMI-1640, Dulbecco’s Phosphate Buffer Saline (DPBS), Fetal bovine serum (FBS), Penicillin-Streptomycin-Neomycin (PSN) antibiotic mixture, Opti-MEM, Lipofectamine LTX, Rabbit polyclonal anti-JNK1 antibody, EnzChek Caspase-3 assay kit and Annexin V-Alexa Fluor 488/ Propidium Iodide (PI) apoptosis assay kit were purchased from Invitrogen (Life Technologies, USA). FITC-Annexin V Apoptosis detection kit for flow cytometry was purchased from BD Biosciences (New Jersey, USA). Dual-Luciferase Reporter Assay System was purchased from Promega (Madison, WI). TNF-α was purchased from ProSpec (Israel). PVDF membrane and Clarity Western ECL substrate were purchased from Bio-Rad (Philadelphia, USA). Rabbit monoclonal antibodies against Poly (ADP-Ribose) polymerase (PARP), Caspase-9, Caspase-3, NF-κB (p65), β-actin, COX IV and anti-rabbit HRP conjugated secondary antibodies were purchased from Cell Signaling Technology (Danvers, MA). Mouse monoclonal antibody against cytochrome c was purchased from Imgenex (San Diego, CA). Rabbit polyclonal anti-p-JNK antibody was purchased from Santa Cruz Biotechnology, Inc. (Dallas, Texas). All other chemicals used were of analytical grade and purchased from Merck (Darmstadt, Germany).

### Cell lines and cell culture

Human colorectal carcinoma (HCT 116), Human colorectal adenocarcinoma (HT-29), Human breast adenocarcinoma (MCF-7), Human embryonic kidney (HEK 293T) and Mouse embryonic fibroblast (NIH 3T3) cell lines were obtained from National Center for Cell Science (NCCS), Pune, Maharashtra, India. HCT 116 and HT-29 cells were cultured in RPMI-1640 medium (Gibco, Life technologies, USA) and MCF-7, HEK 293T and NIH 3T3 cells were cultured in DMEM medium (Gibco, Life technologies, USA). The culture media were supplemented with 10% fetal bovine serum (FBS) and PSN antibiotic solution (Gibco, Life technologies, USA). The cells were kept in a humidified atmosphere of 5% CO_2_ at 37°C. Exponentially growing cells were used for the entire study.

### Preparation and characterization of curcumin-PLGA conjugate

Curcumin was conjugated with Poly (D, L-lactic-co-glycolic acid) (PLGA) via an ester linkage. We prepared conjugate using N, N’-Dicyclohexylcarbodiimide (DCC) and 4-(Di-methylamino) pyridine (DMAP) as activating agents in dichloromethane (DCM). In the resulting suspension, curcumin was added and stirred for 2 h at room temperature. The reaction mixture was lyophilized in freeze dryer (ILSHIN BIOBASE, Korea) and re-crystallized with DCM to obtain curcumin-PLGA conjugate. The resulting conjugate was characterized by Fourier transform infrared spectroscopy (FT-IR) using FT-IR-2000A, ABB Spectrophotometer (ZrCl_2_). For further confirmation of the conjugation, ^1^H NMR spectra of PLGA, native curcumin and curcumin-PLGA conjugate were recorded on Bruker, Avance II (500MHz) Bru spectrometer. Chemical shifts were reported as δ (ppm) relative to Tetramethylsilane (TMS) as a standard. Various shifts in the peaks were interpreted for different groups present in the conjugated system.

### Treatment of curcumin and curcumin-PLGA conjugate

Curcumin and curcumin-PLGA conjugate were freshly prepared in cell culture grade DMSO at a stock concentration of 10 mM each. HCT 116, HT-29, MCF-7, HEK 293T and NIH 3T3 cells were treated with 10 μM and 20 μM of curcumin and curcumin-PLGA conjugate for different time points (6 h, 12 h and 24 h). Similarly, DMSO was used as a vehicle control.

### Determination of IC_50_ value of curcumin-PLGA conjugate

Half maximal inhibitory concentration (IC_50_) value of curcumin and curcumin-PLGA conjugate was determined by MTT assay. Briefly, HCT 116 cells and NIH 3T3 cells were treated with a series of concentrations (2.5 μM, 5 μM, 10 μM, 25 μM, 50 μM, 100 μM, 200 μM) of native curcumin and curcumin-PLGA conjugate for 24 h. The cells were washed twice with DPBS and incubated with 0.5 mg/ml MTT solution for 4 h at 37°C. Thereafter, 0.1 ml of SDS-HCl (10% SDS in 0.01M HCl) was added to each well, mixed thoroughly and allowed for incubation in the dark for 20 min at 37°C. Finally, the absorbance of each well was recorded at 570 nm with a reference wavelength of 650 nm using a multimode microplate reader (SpectraMax M2^e^, Molecular devices, USA). The results are represented in terms of percentage inhibition of cell proliferation compared to that of vehicle control.

### Cell viability and cell proliferation assay

Cell viability was evaluated by trypan blue exclusion assay. HCT 116 cells were treated with 10 μM and 20 μM of curcumin and curcumin-PLGA conjugate for 6 h, 12 h and 24 h. After completion of incubation, cells were harvested and washed once with DPBS. Equal amount of cell suspension was mixed with trypan blue. Subsequently, live and dead cells were counted and percentage of cell death was determined by the following formula (Percentage of cell death = Number of dead cells/Total number of cells x 100). In addition, cell proliferation of HCT 116, HT-29, MCF-7, HEK 293T and NIH 3T3 was examined by MTT assay. The results are represented as percentage of cell proliferation in different treatment group at different time points.

### Colony formation assay

The colony formation assay was performed by crystal violet staining method. In brief, 5 x 10^4^ HCT 116 cells were treated with 10 μM and 20 μM of curcumin and curcumin-PLGA conjugate for 6 h, 12 h and 24 h. Subsequently, cells were trypsinized and 1000 cells were seeded in a 6 well plate (Corning, USA). Cells were allowed to grow for 10 days, until small colonies were visible. The colonies were fixed with methanol and stained with 0.2% crystal violet stain. The ability of a single cell to survive and to grow in a form of colony was considered as plating efficiency. Plating efficiency was defined by the following formula: Percentage plating efficiency (PE) = (Number of colonies formed/Number of cells seeded) x100.

### Cell migration assay

The effects of curcumin and curcumin-PLGA conjugate on cell migration were evaluated by *in vitro* wound scratch assay. Briefly, HCT 116 cells were grown in 6 well culture dishes (Corning, USA). RPMI-1640 medium supplemented with 10% FBS and allowed to reach confluency. A small linear scratch was created in the confluent monolayer using a sterile 200 μl tip. The cells were washed with DPBS to remove cellular debris and subjected to treatment of curcumin and curcumin-PLGA conjugate for 24 h in serum-free media. Thereafter, medium was replaced with fresh serum-free medium and incubated up to 72 h. At the end of incubation, the images of scratches were captured under the DIC filter of an inverted microscope (DP-71, IX81, Olympus, Japan). The area covered by the migrating cells was calculated using Image-Pro MC 6.1 software by comparison of the same fields at 0 h and 72 h. The bar graphs represent the number of migrating cells and distance migrated by cells.

### *In vitro* release of curcumin from curcumin-PLGA conjugate

*In vitro* release study of curcumin from curcumin-PLGA conjugate was carried out by dialysis method. A known concentration (1.0 mg/ml) of curcumin and curcumin-PLGA conjugate was placed in a dialysis bag (10 kDa). The dialysis bag was suspended in RPMI-1640 culture media containing 10% FBS. The entire system was kept at 37 ± 0.5°C with constant stirring of 200 ± 2 rpm. One ml of the solution was withdrawn from the release medium and replaced with fresh media at each time point. The absorbance of curcumin was recorded at 430 nm in multimode microplate reader and the release of curcumin was quantified.

### Cellular uptake assay

Cellular uptake of curcumin and curcumin-PLGA conjugate was examined in HCT 116 cells. Briefly, 1x10^5^ cells were cultured on Poly L-lysine coated coverslips and treated with 10 μM and 20 μM of curcumin and curcumin-PLGA conjugate for 6 h, 12 h and 24 h. The cells were washed once with DPBS and counterstained with DAPI (1 μg/ml). Curcumin exhibit autofluorescence at excitation of 455 nm and emission at 540 nm [24]. Therefore, cellular uptake of curcumin was monitored using GFP filter under fluorescent microscope. The images were analyzed by Image J software (NIH, USA). More than 100 cells from three random fields were examined to show the uptake of curcumin.

### Annexin-V/Propidium Iodide PI staining

The phosphatidyle serine translocation in apoptotic cells was monitored by Annexin V-FITC/ Propidium Iodide staining using an apoptosis detection kit (BioVision, USA). HCT 116 cells were treated with 10 μM and 20 μM of curcumin and curcumin-PLGA conjugate for 6 h, 12 h and 24 h. Thereafter, cells were stained with 5 μl of Annexin-V FITC and 1 μl of Propidium Iodide (PI) for 5 min in dark at room temperature. Annexin V FITC / Propidium Iodide (PI) stained cells were observed under a fluorescent microscope. More than 100 cells from three random fields were taken to examine the apoptotic cell death. All the images were acquired by Image-Pro MC 6.1, (Bethesda, MD, USA) and analyzed by Image J software (NIH, USA). Next, it was also validated by using the Annexin V Alexa Fluor 488 and Proipidium Iodide under Tali image-based cytometer (Life Technologies, USA) as per manufacturer’s instructions. 20 random fields were observed to examine the apoptotic cell death. Further, Annexin V FITC / Propidium Iodide positive cells were monitored by flow cytometry (FACSAria 3, BD Biosciences, San Jose, CA, USA). In brief, 5 x 10^5^ cells were treated with 10 μM and 20 μM of curcumin and curcumin-PLGA conjugate for 12 h (time point is selected on the basis of data obtained in preliminary experiments). Thereafter, cells were collected, washed and re-suspended in 1X Annexin binding buffer followed by the addition of Annexin-V-FITC and Propidium Iodide solution (BD Biosciences, New Jersey, USA). Cells were incubated in the dark for 15 min at room temperature and thereafter subjected to flow cytometric analysis. Data were acquired by BD FACSDiva software (BD Biosciences, San Jose, CA, USA) using standard fluidics, optical and electronic configuration. The light source used was blue laser 488nm with filters, FITC (530/30) and PI (585/42). The FITC and PI channels were compensated with appropriate controls. The Gating on the cell population was set up by FSC/SSC scatter plot. 10,000 events were recorded and analyzed for Annexin-V/Propidium Iodide stain.

### Determination of Caspase 3 activity

Caspase-3 activity was assessed by EnzChek Caspase-3 assay kit according to the manufacturer’s protocol (Life Technologies, USA). In brief, 1 x 10^6^ treated cells were lysed in lysis buffer provided in kit and centrifuged at 5000 rpm for 5 min. Supernatant was collected and concentration of protein was determined by BCA protein estimation kit (Sigma-Aldrich, USA). Next, 100 μg of protein was mixed with 2X substrate solution (Z-DEVD-R110) and incubated for 30 min at 37°C. Finally, fluorescence was recorded at excitation and emission wavelengths of 496 nm and 520 nm respectively, using a multimode microplate reader. The result represents fold increase in activity as compared to vehicle control.

### Analysis of changes in mitochondrial membrane potential (Δψ_m_)

Change in mitochondrial membrane potential (Δψ_m_) was monitored using lipophilic fluorescent probe JC-1. Briefly, 1x10^5^ cells were seeded on poly L-lysine coated coverslips and treated with 10 and 20 μM of curcumin and curcumin-PLGA conjugate for 6 h, 12 h and 24 h. After completion of incubation, residual medium was replaced with DPBS containing JC-1 dye (5 μg/ml) and incubated for 20 min in the dark. Subsequently, cells were counterstained with DAPI for 5 min in the dark and covered with *fluoromount* mounting medium (Sigma-Aldrich, USA). Thereafter, cells were observed under laser scanning confocal microscope (Leica, Germany). In addition, this was also confirmed by quantitative analysis. In brief, 1x10^4^ cells were treated with curcumin and curcumin-PLGA conjugate for mentioned time points and stained with JC-1 dye (5 μg/ml). Fluorescence was recorded at 505 nm excitation and at 527 nm (green fluorescence) and 590 nm (red fluorescence) emission respectively, using a multimode microplate reader. Results were interpreted with ratio of red to green fluorescence representing changes in mitochondrial membrane potential (MMP).

### Determination of Reactive oxygen species (ROS)

The intracellular ROS generation was monitored using a fluorescent dye 2’,7’-Dichlorofluorescein diacetate (H_2_DCFDA). Briefly, 1 x 10^4^ cells were treated with curcumin and curcumin-PLGA conjugate with or without pre-treatment of N-acetyl cysteine (100 μM) for different time points (3 h, 6 h, 9 h and 12 h). Thereafter, cells were incubated with 25 μM H_2_DCFDA for 20 min at 37°C in dark and fluorescence of oxidized product, Dichlorofluorescein (DCF) was recorded at the excitation of 490 nm and emission of 527 nm using a multimode microplate reader. The graphs represent the fluorescence unit of Dichlorofluorescein (DCF).

### Western blotting

Expression of proteins like p65, Caspase-9, Caspase-3, cleaved PARP, p-JNK and JNK were evaluated by western blot analysis. Briefly, 1 x 10^6^ cells were treated with 10 μM and 20 μM of curcumin and curcumin-PLGA conjugate for mentioned time points. Cells were harvested at different time intervals, washed twice with ice-cold DPBS and lysed in lysis buffer (50 mM Tris HCl (pH 8.0), 150 mM NaCl, 1 mM MgCl_2_, 150 mM CaCl_2_, 1 mM PMSF, 1 mM Na-Vanadate, 0.05% Nonidet P-40 containing protease inhibitor cocktail (Roche, USA)) and kept on ice for 30 min followed by disruption and centrifugation at 8,000 rpm (5415R, Eppendorf, Germany) for 10 min at 4°C. The supernatant was collected as a whole cell lysate. Expression of p65 (cytosolic) and cytochrome c (cytosolic and mitochondrial) was determined in sub-cellular fractions. In brief, cells were collected, washed with DPBS (pH-7.4) and lysed in lysis buffer containing 250 mM sucrose, 70 mM KCl, 137 mM NaCl, 4.3 mM Na_2_HPO_4_, 1.4 mM KH_2_PO_4,_ 100 μM PMSF and protease inhibitor cocktail (Roche, Germany) for 5 min on ice. Cells were centrifuged at 1,000 x g for 5 min at 4°C and resulting supernatant was collected as the cytosolic fraction. The remaining pellet was resuspended in mitochondrial lysis buffer containing 50 mM Tris-HCl (pH 7.4), 150 mM NaCl, 2 mM EDTA, 2 mM EGTA, 0.2% (v/v) Triton X-100, 0.3% NP-40, 100 μM PMSF and protease inhibitor cocktail (Roche, Germany) for 5 min on ice. Pellet was disrupted in cell disruptor and centrifuged at 10,000 x g for 5 min and the supernatant was collected as a mitochondrial fraction. The protein concentration was determined by BCA protein estimation kit (Sigma-Aldrich, USA). 50 μg of protein was resolved on 12% SDS-PAGE and transferred to PVDF membrane by wet electroblotting method at 4°C. The membrane was blocked with 5% nonfat milk in TBST (10 mM Tris-HCl (pH 7.4), 0.9% NaCl and 0.05% Tween 20) for 3 h at room temperature followed by overnight incubation of membrane with the primary antibodies: anti-p65(1:250), anti-PARP (1:200), anti-cytochrome c (1:500), anti-caspase-9 (1:200), anti-caspase-3 (1:200), anti-p-JNK (1:200), anti-JNK (1:500), anti-COX IV (1:500) and anti-β-actin (1:500) at 4°C. Membrane was probed with horseradish peroxidase (HRP) conjugated secondary antibodies (1:10,000). Proteins were detected using EZ-ECL kit (Clarity Western ECL substrate, BIO-RAD, USA) according to manufacturer’s instructions and signal was captured on Kodak X-Omat blue film (NEN Life Sciences, Inc., Boston, MA) in the dark.

### Translocation study

The effect of curcumin and curcumin-PLGA conjugate on translocation of p65 between nucleus and cytoplasm was examined by fluorescent microscopy. In brief, 5 x 10^4^ cells were transfected with pEGFP-p65 (gifted by Dr. Johannes Schmid, Med. Uni. Vienna) using Lipofectamine LTX with Plus reagent (Life Technologies, USA), post 24 h of transfection the cells were treated with 20 μM of curcumin and curcumin-PLGA conjugate for different time points (6 h, 12 h and 24 h) followed by exposure of TNF-α (10 ng/ml) for 3 h. The cells were washed with the DPBS and examined under fluorescent microscope. More than 50 cells were examined from three random fields.

### NF-κB Luciferase reporter Assay

NF-κB (p65) activity was monitored by luciferase reporter assay. Briefly, 5 x 10^4^ cells were transiently co-transfected with pGL3b-κB4 and pRL-TK plasmids (gifted by Dr. Susan E. Nozell, Uni. Alabama, USA) using Lipofectamine LTX with Plus reagent (Life Technologies, USA). After 24 h of transfection, cells were treated with 10 μM and 20 μM of curcumin and curcumin-PLGA conjugate for mentioned time points, followed by exposure of TNF-α (10 ng/ml) for 3 h. The assay was performed according to manufacturer’s instructions (Dual-Luciferase Reporter Assay kit, Promega, USA). The results are expressed as the ratio of firefly luciferase activity to that of renilla and normalized to protein concentration.

### Statistical analysis

The data were expressed as mean ± SEM from three independent experiments and analyzed by one way analysis of variance (ANOVA) followed by Student Newman Keuls (SNK) using Sigma Stat 2.0 statistical analysis software. The data were tested for normality by Shapiro-Wilk test prior to ANOVA analysis. Further, multiple comparison in between the groups was performed by SNK test. P value ≤0.05 was considered as statistically significant.

## Results

### Characterization of curcumin and curcumin-PLGA conjugate

**Fig 1:**
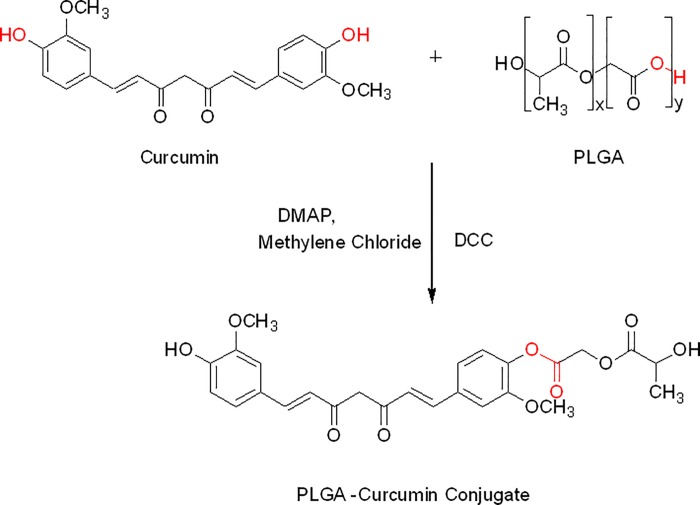
Schematic diagram illustrating the synthesis scheme of curcumin-PLGA conjugate. Curcumin and Poly (D,L-lactic-co-glycolic acid) (PLGA) were conjugated via ester linkage. Poly (D,L-lactic-co-glycolic acid) (PLGA) was activated by N, N’-Dicyclohexylcarbodiimide (DCC) and 4-(Di-methylamino) pyridine (DMAP) in dichloromethane (DCM). The activated PLGA was reacted with curcumin and stirred for 2 h at room temperature. The reaction mixture was lyophilized and re-crystallized with DCM to obtain curcumin-PLGA conjugate.

**Fig 2:**
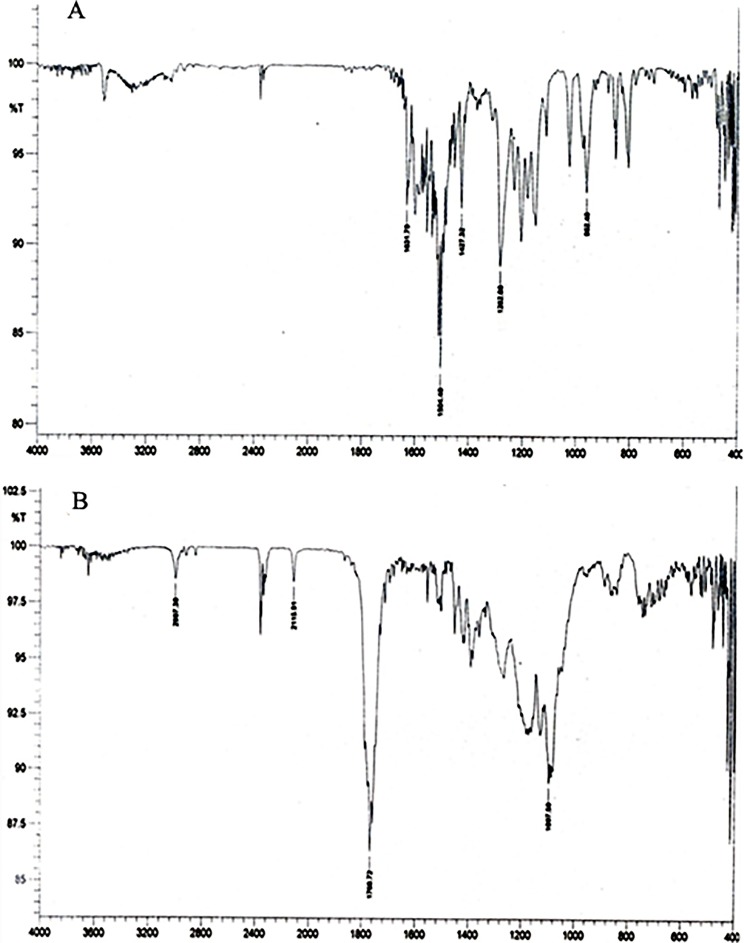
Characterization of curcumin-PLGA conjugate by FT-IR Spectroscopy. FT-IR analysis under FT-IR-2000A, ABB Spectrophotometer (ZrCl_2_) was carried out. The figure represents the spectra of (A) Curcumin and (B) Curcumin-PLGA conjugate.

**Fig 3:**
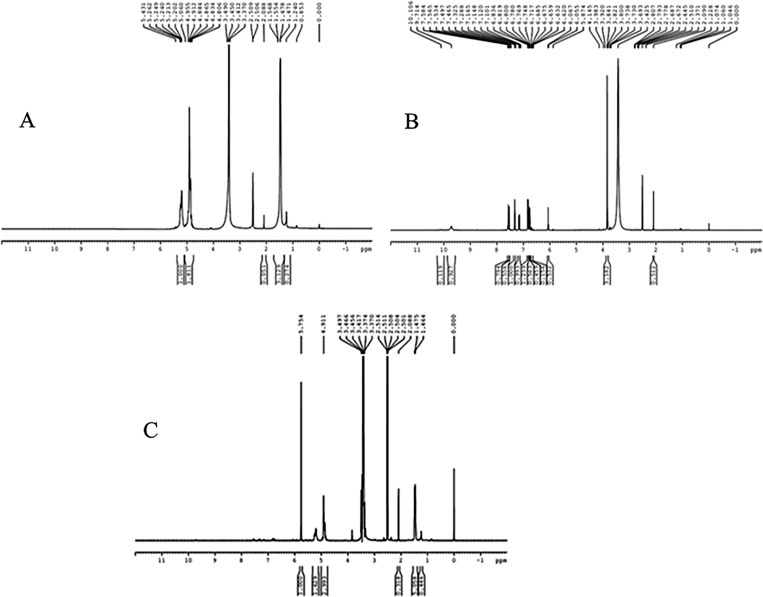
Characterization of curcumin-PLGA conjugate by NMR Spectroscopy. ^1^H NMR analysis was performed on Bruker, Avance II (500MHz) Bru spectrometer. The chemical shifts are reported as δ (ppm) relative to Tetramethylsilane (TMS) spectra of (A) PLGA (B) Curcumin (C) Curcumin-PLGA conjugate

Scheme for conjugation of curcumin with PLGA is shown in Fig. 1. Curcumin and curcumin-PLGA conjugate were analyzed by FT-IR spectroscopy. The changes were recorded with analysis of spectra showing disappearance of broad peaks of hydroxyl in the range of 3500–3200 cm^-1^ and appearance of peak of ester linkage at 1768.72 cm^-1^ in conjugate. It indicates conjugation of curcumin with PLGA (Fig. 2A, 2B). In addition, the NMR spectra showed the following changes in chemical shift values; PLGA- ^1^H NMR (DMSO) δ 5.21 (-CH_2_); δ 1.47 (-CH_3_); δ 4.91 (-CH); δ 2.50 (OH); curcumin- ^1^H NMR (DMSO) δ 7.3 (OH); δ 6.79–6.83 (Ar-H); δ 7.53–7.58 (2H, adjacent to the benzene ring); δ 3.84 (2H, -OCH_3_); δ 6.06 (CO-CH_2_-CO); Conjugate-^1^H NMR (DMSO) δ 4.91 (CO-CH_2_-CO); δ 5.74 (2H, adjacent to the benzene ring); δ 3.74 (2H, -OCH_3_); δ 6.9 (Ar-H). Change in integral values of shifts of secondary -CH_2_ group and disappearance of peaks of the hydroxyl group of curcumin at 7.3 ppm was noted in the conjugated system (Fig. 3A, 3B, 3C). These results confirm the conjugation of curcumin with PLGA.

### Curcumin-PLGA conjugate efficiently inhibit cell proliferation as compared to native curcumin

**Fig 4:**
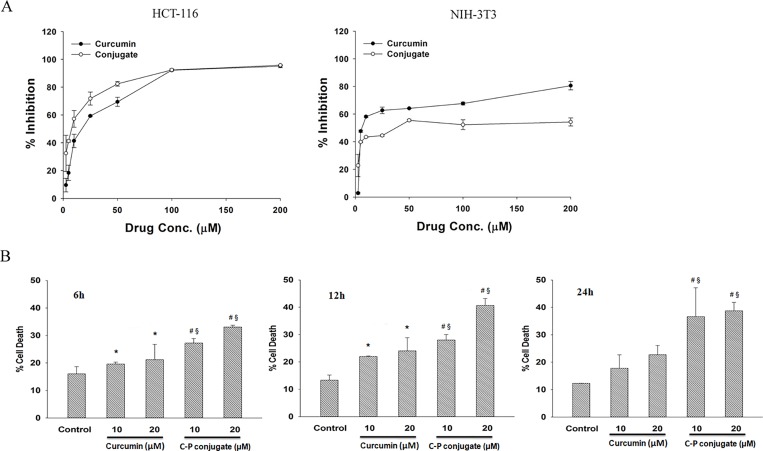
Effect of curcumin and curcumin-PLGA conjugate on cell proliferation. (A) Determination of IC_50_ values of native curcumin and curcumin-PLGA conjugate in HCT 116 and NIH 3T3 cells. HCT 116 and NIH 3T3 cells were treated with the various concentrations (2.5 μM, 5 μM, 10 μM, 25 μM, 50 μM, 100 μM, 200 μM) of curcumin and curcumin-PLGA conjugate for 24 h. 0.5 μl DMSO was used as a vehicle control. Inhibition of cell proliferation was determined by MTT Assay. The obtained values were plotted and the IC_50_ values were determined using SigmaPlot 12.0. (B) Evaluation of cell death by trypan blue exclusion assay. HCT 116 cells were treated with 10 μM and 20 μM of curcumin (C) and curcumin-PLGA conjugate (CP) for 6 h, 12 h and 24 h. Similarly, 0.4 μl DMSO was used as a vehicle control. The cells were harvested, washed, and subjected for trypan blue exclusion assay. The graphs represent the cell death in mentioned concentration of native curcumin (C) and curcumin-PLGA conjugate (CP). Error bars represent mean ±SEM of three independent experiments. Significant difference indicated as *p≤0.05 between untreated and curcumin treated cells; ^#^p≤0.05 between untreated and curcumin-PLGA conjugate treated cells; ^§^p≤0.05 between curcumin and curcumin-PLGA conjugate treated cells (One way ANOVA followed by Student Newman-Keuls multiple comparisons test).

The IC_50_ of native curcumin and curcumin-PLGA conjugate (2.5–200 μM each) were determined from the dose-response curve after 24 h of treatment on HCT 116 cells and NIH 3T3 cells. The IC_50_ value of native curcumin and curcumin-PLGA conjugate was found to be ∼17.22 μM and ∼8.0 μM respectively in HCT 116 cells. However, it was found to be ∼27.59 μM and ∼75.99 μM for native curcumin and curcumin-PLGA conjugate respectively in NIH 3T3 cells (Fig. 4A and S2 Fig.). These results indicate that curcumin-PLGA conjugate at lower concentration effectively inhibited cell proliferation of HCT 116 cells. Based on these results, 10 μM and 20 μM of curcumin and curcumin-PLGA conjugate were selected for further study. Next, cell death was confirmed by Trypan blue and MTT assay. Cell death analysis showed that curcumin-PLGA conjugate efficiently induces cell death in HCT 116 cells from 6–24 h as compared to native curcumin (Fig. 4B). Inhibition of cell proliferation was determined in different cell lines (HCT 116, HT-29, MCF-7, HEK 293T and NIH 3T3). Results showed that curcumin-PLGA conjugate profoundly inhibits cell proliferation as compared to native curcumin in HCT 116, HT-29, MCF-7 and HEK 293T cells at 6–24 h. However, curcumin and curcumin-PLGA conjugate did not show any major change in cell proliferation of NIH 3T3 cells in mentioned concentration (S2 Fig.). Interestingly, cells treated with PLGA did not show any change in cell proliferation in HCT 116 cells (S1 Fig.). Thus, these results indicate that curcumin-PLGA conjugate improves selective inhibition of cell proliferation in cancer cells as compared to native curcumin.

### Curcumin-PLGA conjugate inhibits clonogenic and migration ability

**Fig 5:**
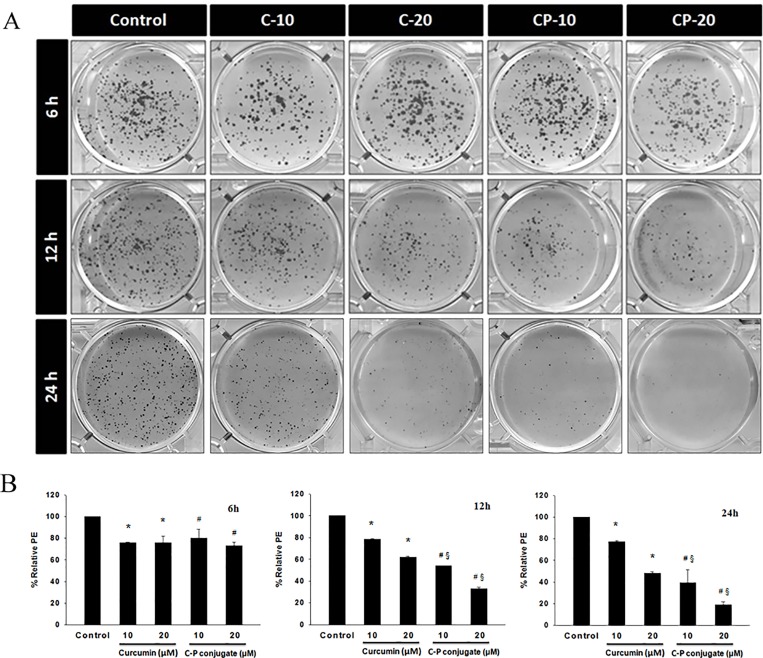
Effect of curcumin and curcumin-PLGA conjugate on the clonogenic ability for cell survival. The colony formation assay was performed by crystal violet staining. HCT 116 cells were treated with 10 μM and 20 μM of curcumin (C) and curcumin-PLGA conjugate (CP) for 6 h, 12 h and 24 h. Similarly, 0.8 μl DMSO was used as a vehicle control. The cells were harvested, washed, and counted. 1000 cells were seeded in 6 well plate and allowed to grow for one week. At the end of incubation, cells were fixed with methanol and stained with 0.2% crystal violet stain. The cells were washed thrice to remove excess stain. (A) Representative image of colony formation. (B) The quantitative representation of colony formation. The plating efficiency was determined from a number of stained colonies. The graphs represent the plating efficiency of cells at mentioned time points and doses. Error bars represent mean ±SEM of three independent experiments. Significant difference indicated as *p≤0.05 between untreated and curcumin treated cells; ^#^p≤0.05 between untreated and curcumin-PLGA conjugate treated cells; ^§^p≤0.05 between curcumin and curcumin-PLGA conjugate treated cells (One way ANOVA followed by Student Newman-Keuls multiple comparisons test).

**Fig 6:**
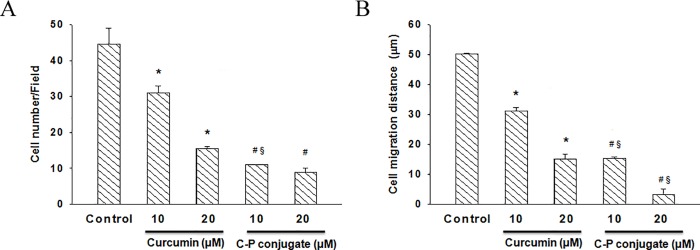
Effects of curcumin and curcumin-PLGA conjugate on the cell migration. The migration ability of HCT 116 cells was monitored by scratch assay. HCT 116 cells were seeded in 6 well plate and allowed to reach the confluency. The scratch was created from center of individual wells on a plate and thereafter cells were treated with 10 μM and 20 μM of curcumin (C) and curcumin-PLGA conjugate (CP) for 6 h, 12 h and 24 h. Similarly, 0.8 μl DMSO was used as a vehicle control. (A) The graph represents the number of migrated cells per field. (B) The graph represents the distance migrated by cells. Error bars represent mean ±SEM of three independent experiments. Significant difference indicated as *p≤0.05 between untreated and curcumin treated cells; ^#^p≤0.05 between untreated and curcumin-PLGA conjugate treated cells; ^§^p≤0.05 between curcumin and curcumin-PLGA conjugate treated cells (One way ANOVA followed by Student Newman-Keuls multiple comparisons test).

We examined the effect of curcumin and curcumin-PLGA conjugate on the clonogenic ability of the cells. This *in vitro* cell survival assay is based on the ability of a single cell to grow into a colony. Colony formation assay revealed that curcumin-PLGA conjugate effectively inhibit colony formation as compared to native curcumin at 12 h and 24 h. The results indicate that curcumin-PLGA conjugate have a low percentage plating efficiency as compared to native curcumin at mentioned time points (Fig. 5A, 5B). Further, we examined the effects of curcumin and curcumin-PLGA conjugate on the migration of HCT 116 cells. Our results demonstrate that curcumin and curcumin-PLGA conjugate inhibit the migration of HCT 116 cells as compared to DMSO control after 72 h. Interestingly, inhibition of cell migration was more prominent in curcumin-PLGA conjugate treated cells as compared to native curcumin (Fig. 6A, 6B), indicating that curcumin-PLGA conjugate effectively inhibits clonogenic and migration ability of HCT 116 cells.

### Curcumin-PLGA conjugate exhibit sustained release and enhanced cellular uptake

**Fig 7:**
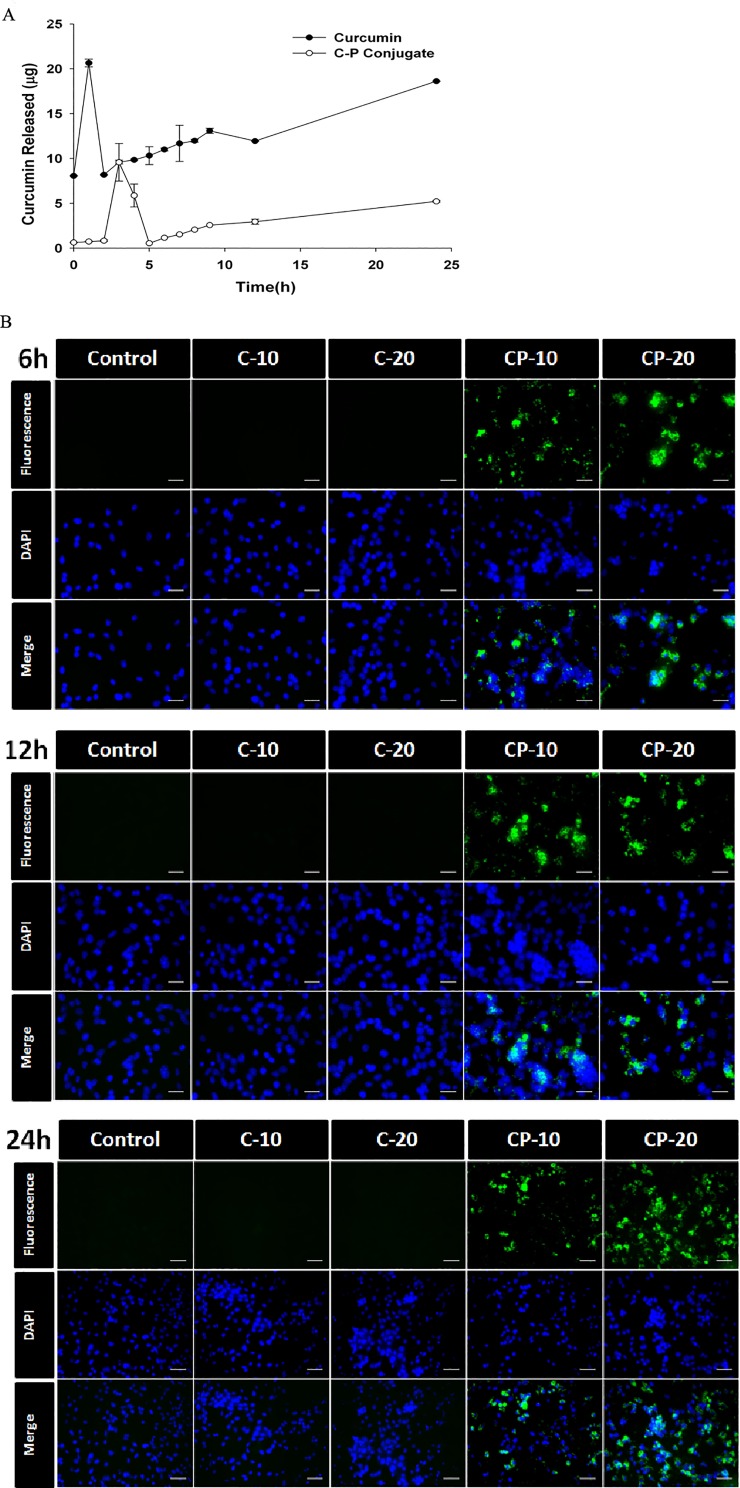
Release study and cellular uptake of curcumin after conjugation with PLGA. (A) *In vitro* release study of native curcumin and curcumin-PLGA conjugate; a known quantity (1 mg/ml) of curcumin or curcumin-PLGA conjugate was placed in dialysis tube and suspended in RPMI 1640 medium with 10% FBS at 37°C. At the end of the incubation period, 1 ml of medium was subjected for the absorbance of curcumin at 430 nm. The released amount of curcumin was quantified using standard curve of curcumin. The plot represents the amount of curcumin released at mentioned time points. (B) Qualitative analysis of cellular uptake of curcumin was examined by its intrinsic property of autofluorescence at GFP filter under fluorescence Microscope. HCT 116 cells were treated with 10 μM and 20 μM of curcumin (C) and curcumin-PLGA (CP) conjugate for 6 h, 12 h and 24 h. Subsequently, the cells were counterstained with DAPI and analyzed under a fluorescence microscope. Scale bar represents 20 μm.

We examined the release of curcumin from curcumin-PLGA conjugate *in vitro*. We found that curcumin PLGA conjugate exhibits slow release (Fig. 7A). Further, cellular uptake of curcumin and curcumin-PLGA conjugate was examined by utilizing its intrinsic fluorescence property. It was observed that the cells were treated with curcumin-PLGA conjugate showed intense green fluorescence within the cells, while those treated with native curcumin did not show any fluorescence at the mentioned time points (Fig. 7B). These results support our hypothesis that conjugation of curcumin with PLGA improves its retention and cellular uptake in HCT 116 cells.

### Curcumin-PLGA conjugate demonstrates enhanced apoptotic cell death

**Fig 8:**
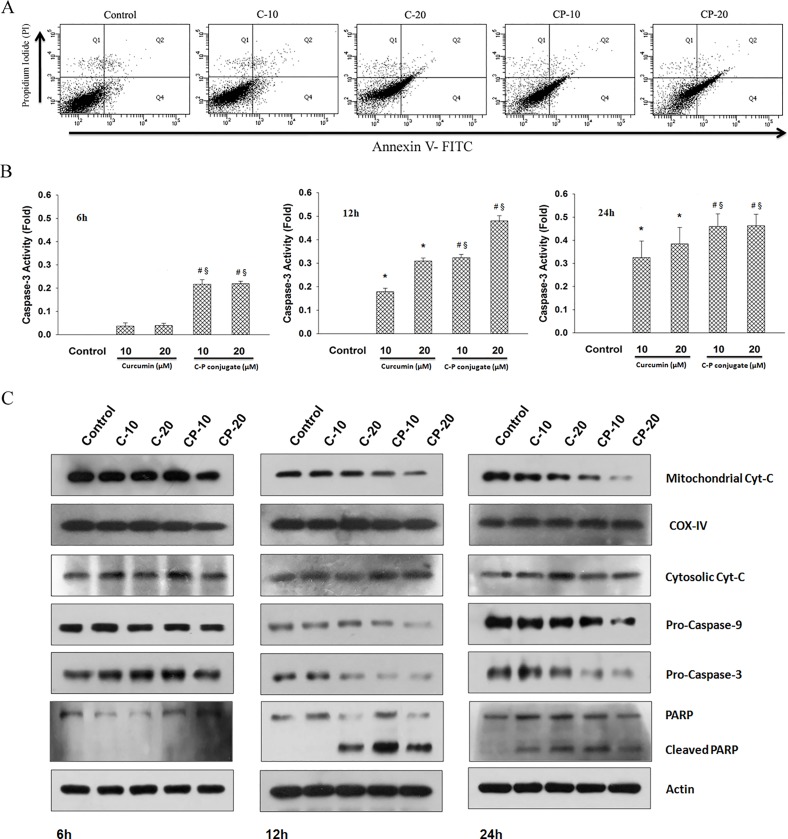
Execution of apoptotic cell death by curcumin and curcumin-PLGA conjugate. (A) Determination of apoptosis by flow cytometery. HCT 116 cells were treated with 10 μM and 20 μM of curcumin (C) and curcumin-PLGA (CP) conjugate for 12 h. Similarly, 8 μl DMSO was used as a vehicle control. Subsequently, the cells were harvested, washed with DPBS and stained with Annexin-V FITC/ Propidium Iodide (PI). Annexin-V and Propidium Iodide stained cells were acquired under the FACSAria flow cytometer. The obtained data were analyzed by FACSDiva software. Cells were categorized into four groups based on dye uptake. The quadrant (Q) showing Q 1: PI positive, Q 2: Annexin V/PI positive, Q 3: Annexin V/PI negative and Q 4: Annexin V positive. (B) Caspase-3 activity was determined by EnzChek Caspase-3 Assay kit. HCT 116 cells were treated with 10 μM and 20 μM of curcumin (C) and curcumin-PLGA (CP) conjugate for 6 h, 12 h and 24 h. Similarly, 0.8 μl DMSO was used as a vehicle control. The cells were lysed and 100 μg of protein was subjected for caspase-3 activity. Graphs represent the fold increase in activity. Error bars represent mean ±SEM from three independent experiments. Significant difference indicated as *p≤0.05 between untreated and curcumin treated cells; ^#^p≤0.05 between untreated and curcumin-PLGA conjugate treated cells; ^§^p≤0.05 between curcumin and curcumin-PLGA conjugate treated cells (One way ANOVA followed by Student Newman-Keuls multiple comparisons test). (C) Western blots of apoptosis regulatory proteins: procasapse-9, procaspase-3, PARP and cytochrome c (cytosolic and mitochondrial fractions). COX IV and β-actin were used as a loading control.

Curcumin is well known to induce apoptotic cell death [25]. Hence, we examined the potential of curcumin-PLGA conjugate for induction of apoptotic cell death. Both qualitative and quantitative analysis revealed a gradual increase of Annexin V and Propidium Iodide stain in curcumin-PLGA conjugate treated cells as compared to native curcumin at different time points (S3 and S4 Figs.). Flow cytometric analysis also confirmed that HCT 116 cells treated with curcumin-PLGA conjugate have optimally higher percentage of apoptotic cell death as compared to native curcumin at 12 h (Fig. 8A). As proteolytic activation of caspases is a hallmark of apoptotic death signaling, we examined the activity of executioner caspase-3. We observed that the activity was about two fold higher in curcumin-PLGA conjugate treated cells as compared to native curcumin (Fig. 8B). The results of western blotting showed that curcumin-PLGA conjugate downregulates the expression of mitochondrial cytochrome c as compared to native curcumin. Meanwhile, the expression of cytosolic cytochrome c was found to increase. Moreover, reduced level of pro-caspase-3 and pro-caspase-9 was observed (Fig. 8C). Thus, these results clearly indicate that curcumin PLGA conjugate efficiently executes apoptosis by releasing cytochrome c from mitochondria, which induces activation of downstream caspases for apoptotic cell death.

### Curcumin-PLGA conjugate reduces mitochondrial membrane potential for induction of cell death

**Fig 9:**
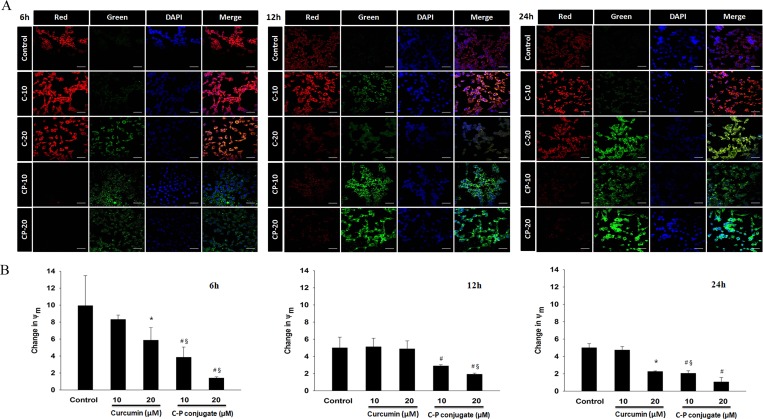
Changes in mitochondrial membrane potential. The mitochondrial membrane potential (MMP) was monitored by the JC-1 dye. HCT 116 cells were treated with 10 μM and 20 μM of curcumin (C) and curcumin-PLGA conjugate (CP) for 6 h, 12 h and 24 h. Similarly, 1 μl DMSO was used as a vehicle control. Thereafter, the cells were washed and incubated with JC-1 dye for 15 min and subsequently counterstained with DAPI. (A) Qualitative analysis of changes in MMP performed by confocal microscopy. Scale bar represents 20 μm. (B) Qualitative analysis of changes in MMP examined by quantification of fluorescence of the JC-1 dye (excitation at 505 nm and emission at 527 nm and 590 nm). The ratio of red fluorescence to green fluorescence was considered as change in mitochondrial membrane potential. Error bars represent mean ±SEM from three independent experiments. Significant difference indicated as *p≤0.05 between untreated and curcumin treated cells; ^#^p≤0.05 between untreated and curcumin-PLGA conjugate treated cells; ^§^p≤0.05 between curcumin and curcumin-PLGA conjugate treated cells (One way ANOVA followed by Student Newman-Keuls multiple comparisons test).

The early loss of mitochondrial membrane potential is an indicator of apoptotic cell death. Here, we again confirmed the mitochondria dependent induction of cell death by observing changes in mitochondrial membrane potential using JC-1 dye under confocal microscope. In healthy cells, JC-1 dye forms complexes known as J-aggregates and stay within mitochondria which emits red fluorescence. On the other hand, in apoptotic or unhealthy cells due to loss of mitochondrial membrane potential it remains in monomeric form in the cytoplasm, which emits green fluorescence. Our results showed a progressive change in mitochondrial membrane potential in curcumin-PLGA treated cells as compared to native curcumin (Fig. 9A). In addition, quantitative analysis obtained by spectrofluorometric analysis revealed a similar result (Fig. 9B). Ratio of red fluorescence to green fluorescence was lower in curcumin-PLGA conjugate treated cells as compared to native curcumin (Fig. 9B).

### Curcumin-PLGA conjugate promotes ROS dependent JNK mediated cell death

**Fig 10:**
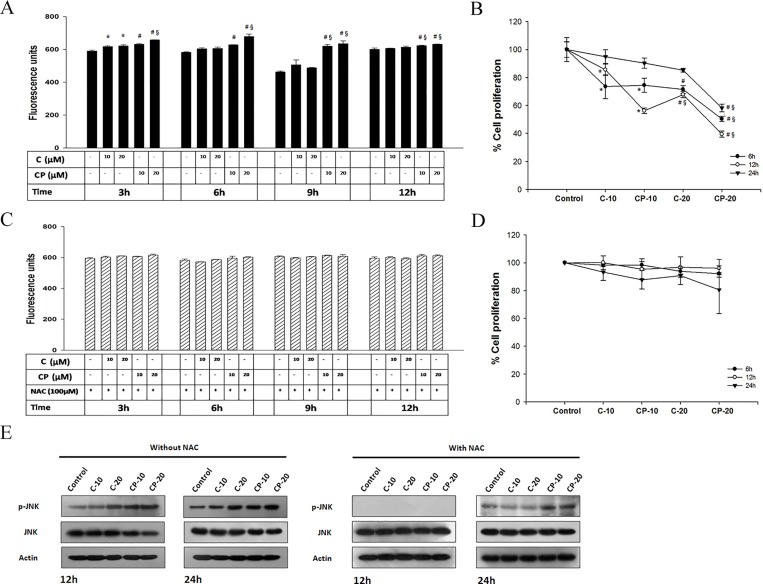
Effect of Curcumin and curcumin-PLGA conjugate on ROS generation and JNK activation. HCT 116 cells were treated with 10 μM and 20 μM of curcumin (C) and curcumin-PLGA conjugate (CP) for 3 h, 6 h, 9 h and 12 h. Similarly, 0.4 μl DMSO was used as a vehicle control. Thereafter, the cells were washed and incubated with H_2_DCFDA dye for 20 min. The liberation dichlorofluorescein (DCF) fluorescence was recorded under multi-mode plate reader. Graphs represent the fluorescence unit of dichlorofluorescein (DCF). (A) Graph represents a level of ROS in the absence of N-acetyl cysteine (NAC). (B) Percent inhibition of cell proliferation determined by MTT assay in the absence of NAC. (C) Graph represents a level of ROS in the presence of N-acetyl cysteine (NAC). (D) Percent inhibition of cell proliferation determined by MTT assay in the presence of NAC. Error bars represent mean ±SEM from three independent experiments. Significant difference indicated as *p≤0.05 between untreated and curcumin treated cells; ^#^p≤0.05 between untreated and curcumin-PLGA conjugate treated cells; ^§^p≤0.05 between curcumin and curcumin-PLGA conjugate treated cells (One way ANOVA followed by Student Newman-Keuls multiple comparisons test). (E) Western blots of phosphorylated JNK and JNK in presence and absence of NAC. Cells were pretreated with NAC for 3 h and thereafter treated with 10 μM and 20 μM of curcumin (C) and curcumin-PLGA conjugate (CP) for 12 h and 24 h.

Here, we examined the effect of curcumin-PLGA on ROS generation and JNK mediated cell death. Results obtained from quantitative analysis showed that curcumin-PLGA conjugate moderately increases intracellular ROS as compared to native curcumin (Fig. 10A). However, in presence of NAC (inhibitor of ROS) no major change was observed in generation of intracellular ROS (Fig. 10C). Next, ROS dependent JNK activation was confirmed by western blotting of phosphorylated JNK and JNK in presence and absence of NAC. The HCT 116 cells treated with curcumin-PLGA conjugate show elevated level of phosphorylated JNK as compared to native curcumin at 12 h and 24 h. However, in presence of NAC, the expression of phosphorylated JNK was downregulated at 12 h and was minimal at 24 h in both curcumin and curcumin-PLGA conjugate treated cells (Fig. 10E). These results confirm that the curcumin PLGA conjugate promotes ROS dependent JNK mediated apoptotic cell death in HCT 116 cells. Further, the cell death was validated in presence and absence of NAC by MTT assay. Results showed that potential of curcumin-PLGA conjugate on cell death was inhibited in the presence of ROS scavenger, NAC (Fig. 10B, 10D). Collectively, our results indicae that curcumin-PLGA conjugate efficiently induces ROS dependent JNK mediated cell death.

### Curcumin-PLGA conjugate inhibits NF-κB activation

**Fig 11:**
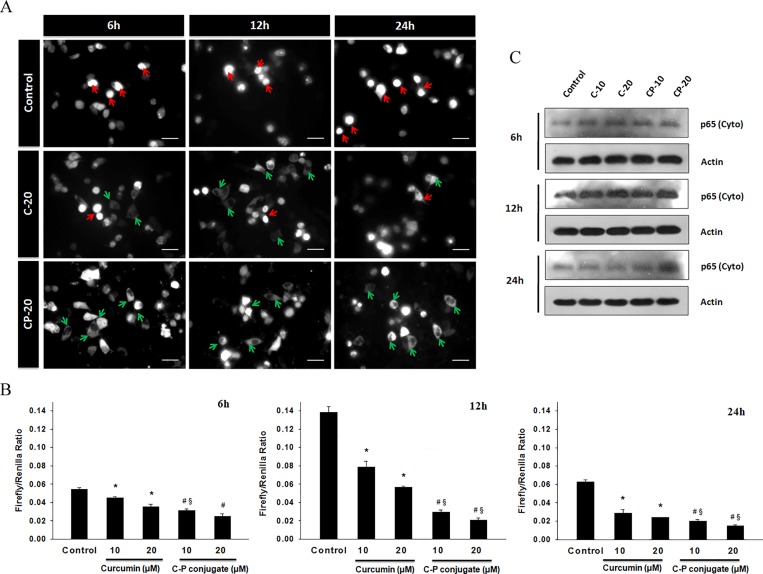
Effects of curcumin and curcumin-PLGA conjugate on NF-κB activity. HCT 116 cells were transfected with GFP-p65 (NF-κB) for 24 h and treated with 20 μM of curcumin (C) and curcumin-PLGA conjugate (CP) for 6 h, 12 and 24 h. Subsequently, after completion of incubation the cells were washed and exposed to TNF-α (10 ng/ml) for 3 h and analyzed under a fluorescence microscope. (A) Representative image of GFP-p65 localization in HCT 116 cells. Red arrow shows translocation of p65 in the nucleus and green arrow shows cytosolic p65. Scale bar represents 20 μm. (B) Analysis of transcriptional activity of NF-κB. HCT 116 cells were transiently transfected with pGL3b-κB4 and pRL-TK plasmids for 24 h and treated with curcumin and curcumin-PLGA conjugate for 6 h, 12 h and 24 h. Subsequently, the cells were retreated with TNF-α for 3 h. After completion of incubation, the cells were subjected for NF-κB activity using the luciferase activity assay kit. Graphs represent the NF-κB activity in terms of the ratio of luminescence of firefly luciferase activity to luminescence of renilla luciferase activity. Error bars represent mean ±SEM of three independent experiments. Significant difference indicated as *p≤0.05 between untreated and curcumin treated cells; ^#^p≤0.05 between untreated and curcumin-PLGA conjugate treated cells; ^§^p≤0.05 between curcumin and curcumin-PLGA conjugate treated cells (One way ANOVA followed by Student Newman-Keuls multiple comparisons test). (C) Cytosolic expression of p65: p65 (cyto) examined by western blotting.

NF-κB, a transcription factor plays vital role in the regulation of proliferation, apoptosis and survival of the cell. NF-κB mainly sequestered in the cytoplasm in an inactive form, but upon activation it translocates to the nucleus for activation of various sets of genes [26]. As several earlier reports highlighted that curcumin inhibits p65 activation [27], NF-κB activation was monitored by p65-GFP translocation. We observed that curcumin-PLGA conjugate treated cells prominently retained p65 in cytoplasm and the translocation to the nucleus was inhibited even in presence of TNF-α (Fig. 11A). Activity of NF-κB was also examined by luciferase reporter assay. It was found that curcumin-PLGA conjugate inhibits NF-κB activation more effectively as compared to native curcumin (Fig. 11B). Moreover, cytosolic retention of p65 in treated cells was also confirmed by western blotting. The result showed elevated expression of p65 in cytosolic fraction (Fig. 11C). Thus, our data suggest that curcumin-PLGA conjugate efficiently inhibits NF-κB activation and protects from cell survival.

## Discussion

Curcumin is a bioactive component of *Curcuma longa* and well identified for its medicinal properties since ancient history [28]. A variety of functional groups, including the β-diketo group, carbon–carbon double bonds and phenyl rings containing varying amounts of hydroxyl and methoxy substituents exhibits its anti-oxidant properties. Earlier reports suggest that the keto groups, carbon–carbon double bonds and phenolic methoxy substituent are important for its anti-cancer activity [29, 30]. Since last decade, curcumin has shown incredible biological feature but instability at physiological pH and low bioavailability limits its clinical applications [19, 31]. Several studies have suggested that poor bioavailability of curcumin is due to its early biotransformation and metabolism [32, 33]. To overcome such limitations, several approaches have been used to improve its therapeutic efficacy. In the present study, we conjugated the curcumin with biodegradable polymer PLGA to improve its stability and hence bioavailability. Conjugation of polymeric nanoparticles with ligands or molecules could also enable drug delivery in a spatially and temporally controlled manner, which may further enhance the therapeutic efficacy of drugs and reduce their toxicity [34]. Here, we have conjugated the curcumin with PLGA through an ester bond formation at a phenolic hydroxyl group of curcumin that might result in an increase in its stability. We speculated that ester linkage at the phenolic group on curcumin would be hydrolyzed by cytosolic esterases, thereby releasing native curcumin gradually inside the cells, which can elicit its activity. In our study, we analyzed anti-proliferative effect, cellular uptake and the ability to induce apoptotic cell death of curcumin-PLGA conjugate and native curcumin on human colon carcinoma HCT 116 cells. In order to acquire experimental evidences, initially we confirmed the conjugation of curcumin with PLGA by FT-IR and NMR spectroscopy. The disappearance of broad peaks of hydroxyl in the range of 3500–3200 cm^-1^ and appearance of peak corresponding to an ester linkage at 1768.72 cm^-1^ confirms conjugation of curcumin with PLGA. In addition, NMR data also revealed a change in integral values of shifts of secondary -CH_2_ group and disappearance of peaks of the hydroxyl group of curcumin at 7.3 ppm that confirmed the conjugation.

First, we examined the inhibitory concentration of native curcumin and curcumin-PLGA conjugate on HCT 116 cells and NIH 3T3 cells. The IC_50_ value of native curcumin was observed ∼17.22 μM and ∼ 8.0 μM for curcumin-PLGA conjugate in HCT 116 cells. These data showed that IC_50_ value of curcumin-PLGA conjugate is two-fold lower as compared to native curcumin. Interestingly, the IC_50_ value of native curcumin and curcumin-PLGA conjugate was ∼27.59 μM and ∼75.99 μM respectively in NIH 3T3 cells. The results obtained from this study suggest that conjugation of curcumin with PLGA reduces cytotoxic response and enhances the efficacy of curcumin. Therefore, we selected 10 μM and 20 μM concentrations of curcumin and curcumin-PLGA conjugate for further study. Our observation is consistent with an earlier finding which highlighted that 20–30 μM of curcumin treatment inhibits cell proliferation and induces apoptosis in various cancer cells [25, 35]. Further, we evaluated the anti-proliferative effect of curcumin-PLGA conjugate in various cell lines i.e. HT-29, MCF-7, HEK 293T and NIH 3T3 cells. We observed that the cells were treated with curcumin-PLGA conjugate show a significant inhibition of cell proliferation in time and dose dependent manner as compared to native curcumin. Further, we validated the effect of curcumin-PLGA conjugate and native curcumin on cell survival by colony formation and cell migration assay. Our results demonstrate that curcumin-PLGA conjugate inhibits colony formation and cell migration in HCT 116 cells. From these findings we infer that curcumin-PLGA conjugate potentially inhibits cell proliferation as compared to native curcumin.

The instability and low bioavailability of curcumin is major curtail for its pharmacological efficacy. Moreover, previous reports suggested that drug conjugated with carrier molecules imparts sustained release of the drug to improve its biological response [36, 37]. In our study, we found that in the curcumin conjugated with PLGA exhibits slower release as compared to native curcumin at physiological pH. Next, the cellular uptake of native curcumin and curcumin-PLGA conjugate was revealed by its intrinsic autofluorescence property under fluorescent microscope. We observed that cells treated with curcumin-PLGA conjugate exhibit intense fluorescence, while no fluorescence was observed in native curcumin treated cells at 6–24 h. Thus, these results indicated that conjugation of curcumin with PLGA effectively protected from rapid metabolism and degradation. Interestingly, above findings demonstrate that conjugation of curcumin with PLGA improves cellular uptake and stability of curcumin.

Curcumin is known to induce apoptotic cell death in various types of cancer [25, 38, 39]. The molecular mechanism of apoptotic cell death induced by curcumin was mostly demonstrated by mitochondria-dependent pathway [25, 40]. In this study, we investigated the mechanism by which curcumin-PLGA conjugate manifest apoptotic potential in HCT 116 cells. Interestingly, we noticed that curcumin-PLGA conjugate induces rapid loss of mitochondrial membrane potential to activate apoptotic signaling as compared to native curcumin. Furthermore, the western blot analysis of apoptotic proteins revealed that the curcumin-PLGA conjugate efficiently induce apoptosis by release of cytochrome c and activation of downstream cascade of caspases i.e. pro-caspase-9 and pro-caspase-3. Curcumin is also known to exhibits ROS production and modulation of JNK signaling to execute apoptosis in cancer cells [41, 42]. Previous study highlighted that curcumin perturbs cellular redox potential by upregulation of ROS which leads to JNK activation and loss of mitochondrial membrane potential [43]. In our study, we found that curcumin-PLGA conjugate upregulates intracellular level of ROS as compared to native curcumin, which leads to phosphorylation of JNK and endorsement of cell death. However, in the presence of NAC (inhibitor of ROS), phosphorylation of JNK and cell death was abrogated. Moreover, curcumin is known to inhibit multiple signaling pathways and NF-κB activation in cancer cells [44, 45]. Here, we investigated the effect of curcumin-PLGA conjugate on NF-κB activation. Our result showed inhibition of TNF-α induced cytosolic to nuclear translocation of p65 in HCT 116 cells. In addition, Western blot and the luciferase activity assay for p65 revealed similar results. Thus, these results suggest that curcumin PLGA conjugate attenuates NF-κB activation.

**Fig 12:**
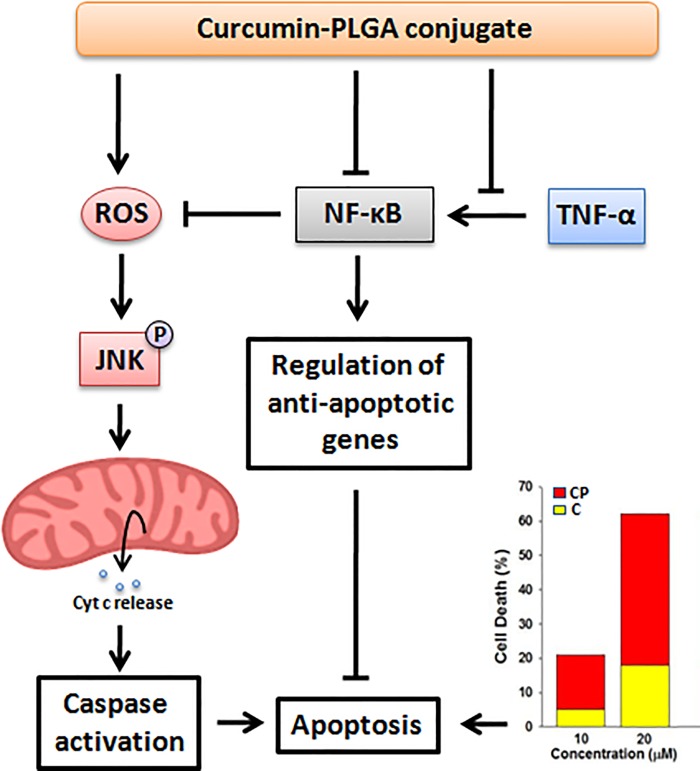
Schematic diagram illustrate anti-proliferative and apoptotic potential of curcumin-PLGA conjugate.

In conclusion, the present study demonstrates that the conjugation with PLGA substantially enhances the biological activity of curcumin by promoting its cellular uptake and stability without altering its inherent properties and pleiotropic effects. In addition, curcumin conjugated with PLGA inhibits cell proliferation and impairs cell survival by modulating apoptotic signaling more efficiently as compared to native curcumin (Fig. 12). Thus, we can suggest that conjugation of anti-cancer agents with biodegradable polymers may offer a new approach for anti-cancer therapy.

## Supporting Information

S1 Fig Effect of PLGA on cell proliferation of HCT 116 cells. HCT 116 cells were treated with 10 μM and 20 μM of PLGA for 6 h, 12 h and 24 h. Similarly, 0.4 μl DMSO was used as a vehicle control. Cell proliferation was examined by MTT assay. The graphs represent percentage of cell proliferation. No significant difference was observed in PLGA treated cells as compared to control. Error bars represent mean ±SEM of three independent experiments. P-10 (10 μM PLGA), P-20 (20 μM PLGA).
(TIF)

S2 Fig Effects of curcumin and curcumin-PLGA conjugate on cell proliferation of HCT 116, HT-29, HEK 293T, MCF-7 and NIH 3T3 cells. HCT 116, HT-29, HEK 293T, MCF-7 and NIH 3T3 cells were treated with 10 μM and 20 μM of curcumin and curcumin-PLGA conjugate for 6 h, 12 h and 24 h. Similarly, 0.4 μl DMSO was used as a vehicle control. Cell proliferation was evaluated by MTT assay. The bar graphs represent percentage of cell proliferation at mentioned time and concentrations. The significant reduction in cell proliferation was observed in curcumin-PLGA conjugate treated HCT 116, HT-29, HEK 293T and MCF-7 cells as compared to native curcumin. No significant difference was observed in proliferation of NIH 3T3 cells (Normal mouse embryonic fibroblast cells). Error bars represent mean ±SEM of three independent experiments. Significant difference indicated as *p≤0.05 between untreated and curcumin treated cells; ^#^p≤0.05 between untreated and curcumin-PLGA conjugate treated cells; ^§^p≤0.05 between curcumin and curcumin-PLGA conjugate treated cells (One way ANOVA followed by Student Newman-Keuls multiple comparisons test).
(TIF)

S3 Fig Qualitative analysis of effects of curcumin and curcumin-PLGA conjugate on apoptotic cell death. The HCT 116 cells were treated with 10 μM and 20 μM of curcumin and curcumin-PLGA conjugate for 6 h, 12 h and 24 h. Similarly, 1 μl DMSO was used as a vehicle control. Thereafter, the cells were stained with Annexin-V FITC for 10 min and subsequently stained with Propidium Iodide for 5 min in the dark at room temperature. Annexin-V FITC/ Propidium Iodide stained cells were observed under a fluorescent microscope. Scale bar represents 20 μm.
(TIF)

S4 Fig Quantitative analysis of effects of curcumin and curcumin-PLGA conjugate on apoptotic cell death. The HCT 116 cells were treated with 10 μM and 20 μM of curcumin and curcumin-PLGA conjugate for 6 h, 12 h and 24 h. Similarly, 2 μl DMSO was used as a vehicle control. Thereafter, apoptotic cell death was validated by using Annexin V-Alexa Fluor 488/ Propidium Iodide (PI) apoptosis detection kit, under automated image-based cytometer (Tali, Life Technologies, USA) according to manufacturer’s instructions. The cells were observed from 20 random fields for validation. (A) Representative images of Annexin-V/Propidium Iodide (PI) stained cells are shown in different panels. (B) The bar graphs represent percentage of Annexin-V and Propidium Iodide stained cells for late apoptosis. Error bars represent mean ±SEM of three independent experiments. Significant difference indicated as *p≤0.05 between untreated and curcumin treated cells; ^#^p≤0.05 between untreated and curcumin-PLGA conjugate treated cells; ^§^p≤0.05 between curcumin and curcumin-PLGA conjugate treated cells (One way ANOVA followed by Student Newman-Keuls multiple comparisons test).
(TIF)
