# Supplementary figures and images for: Curcumin Conjugated with PLGA Potentiates Sustainability, Anti-Proliferative Activity and Apoptosis in Human Colon Carcinoma Cells
Source: PLoS One. 2015 Feb 18;10(2):e0117526. doi: 10.1371/journal.pone.0117526 (PMC4334672; doi:10.1371/journal.pone.0117526)

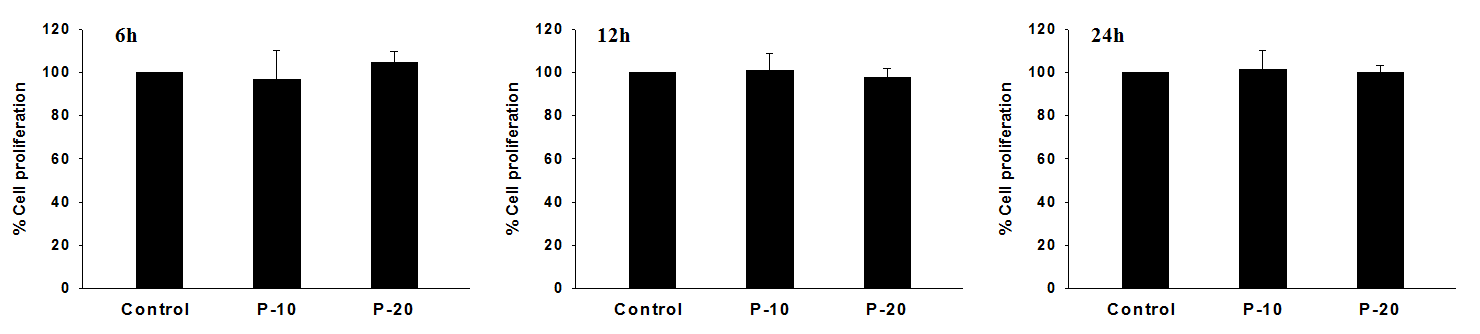

Supplement: S1 Fig — HCT 116 cells were treated with 10 μM and 20 μM of PLGA for 6 h, 12 h and 24 h. Similarly, 0.4 μl DMSO was used as a vehicle control. Cell proliferation was examined by MTT assay. The graphs represent percentage of cell proliferation. No significant difference was observed in PLGA treated cells as compared to control. Error bars represent mean ±SEM of three independent experiments. P-10 (10 μM PLGA), P-20 (20 μM PLGA). (TIF) [file pone.0117526.s001.tif]

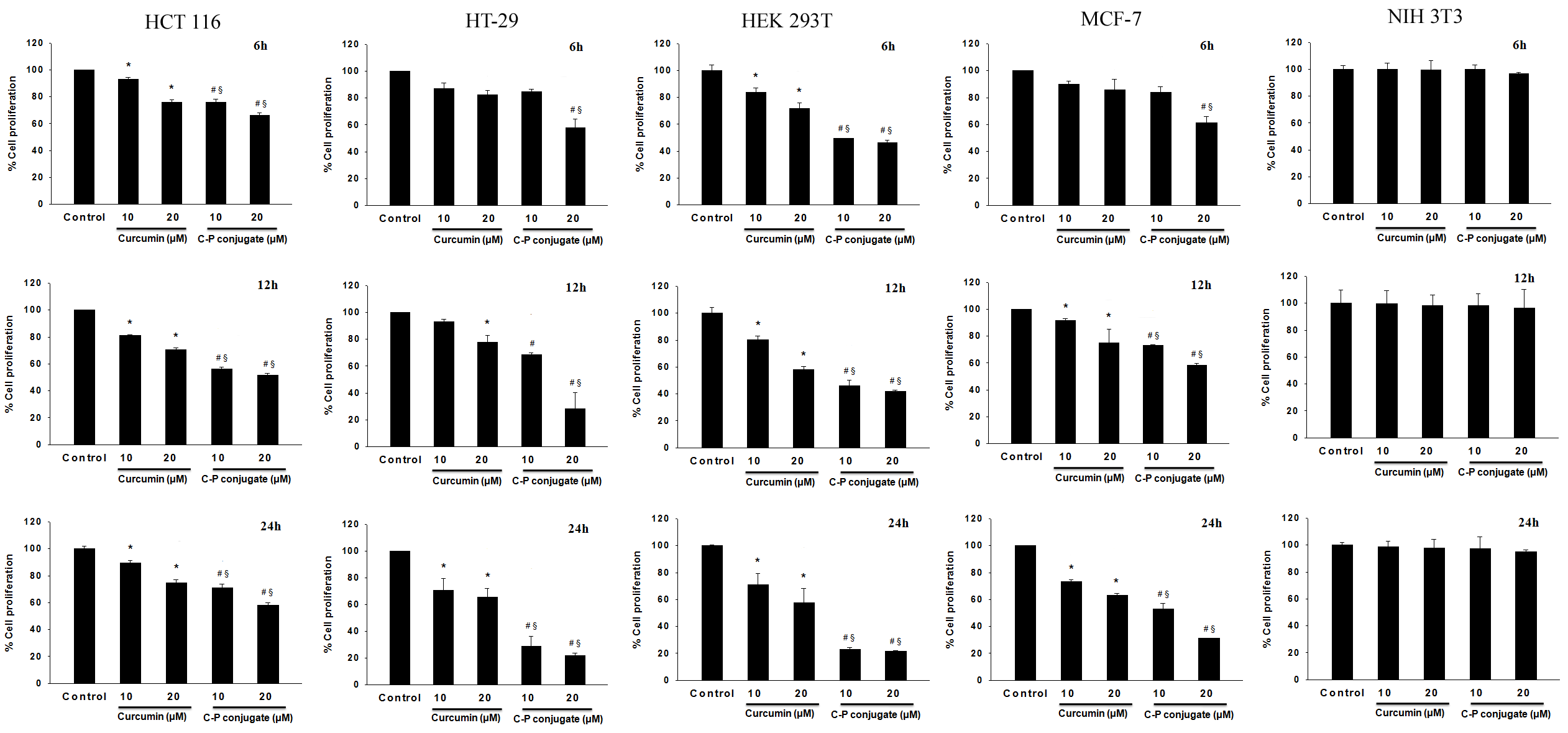

Supplement: S2 Fig — HCT 116, HT-29, HEK 293T, MCF-7 and NIH 3T3 cells were treated with 10 μM and 20 μM of curcumin and curcumin-PLGA conjugate for 6 h, 12 h and 24 h. Similarly, 0.4 μl DMSO was used as a vehicle control. Cell proliferation was evaluated by MTT assay. The bar graphs represent percentage of cell proliferation at mentioned time and concentrations. The significant reduction in cell proliferation was observed in curcumin-PLGA conjugate treated HCT 116, HT-29, HEK 293T and MCF-7 cells as compared to native curcumin. No significant difference was observed in proliferation of NIH 3T3 cells (Normal mouse embryonic fibroblast cells). Error bars represent mean ±SEM of three independent experiments. Significant difference indicated as *p≤0.05 between untreated and curcumin treated cells; #p≤0.05 between untreated and curcumin-PLGA conjugate treated cells; §p≤0.05 between curcumin and curcumin-PLGA conjugate treated cells (One way ANOVA followed by Student Newman-Keuls multiple comparisons test). (TIF) [file pone.0117526.s002.tif]

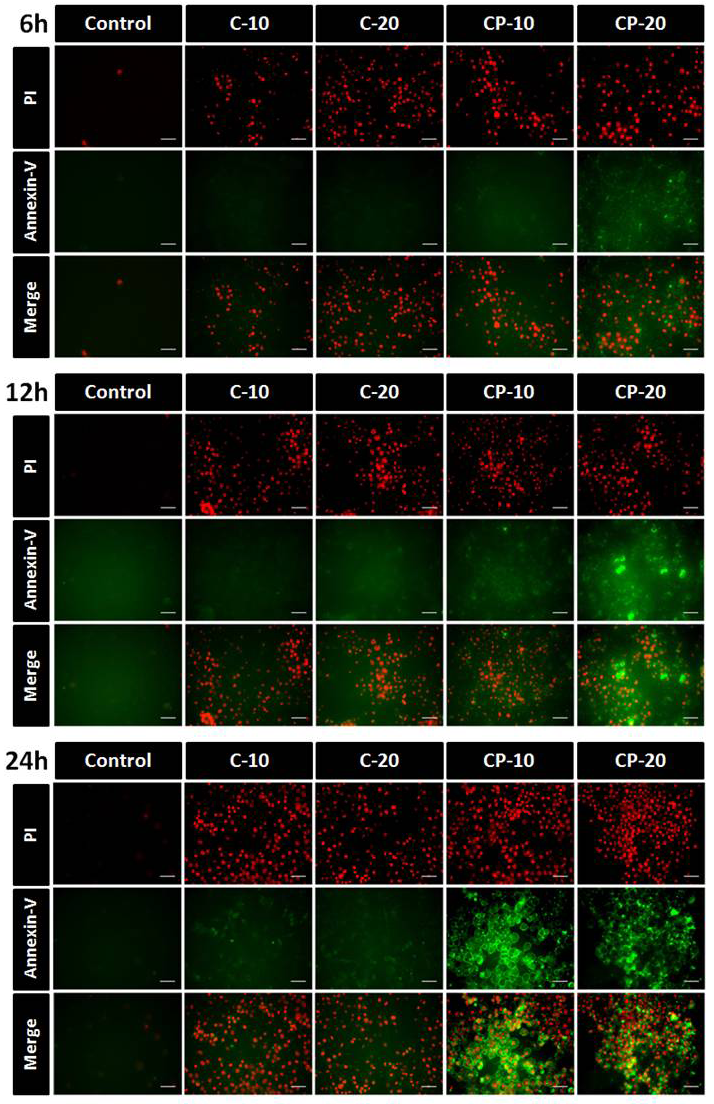

Supplement: S3 Fig — The HCT 116 cells were treated with 10 μM and 20 μM of curcumin and curcumin-PLGA conjugate for 6 h, 12 h and 24 h. Similarly, 1 μl DMSO was used as a vehicle control. Thereafter, the cells were stained with Annexin-V FITC for 10 min and subsequently stained with Propidium Iodide for 5 min in the dark at room temperature. Annexin-V FITC/ Propidium Iodide stained cells were observed under a fluorescent microscope. Scale bar represents 20 μm. (TIF) [file pone.0117526.s003.tif]

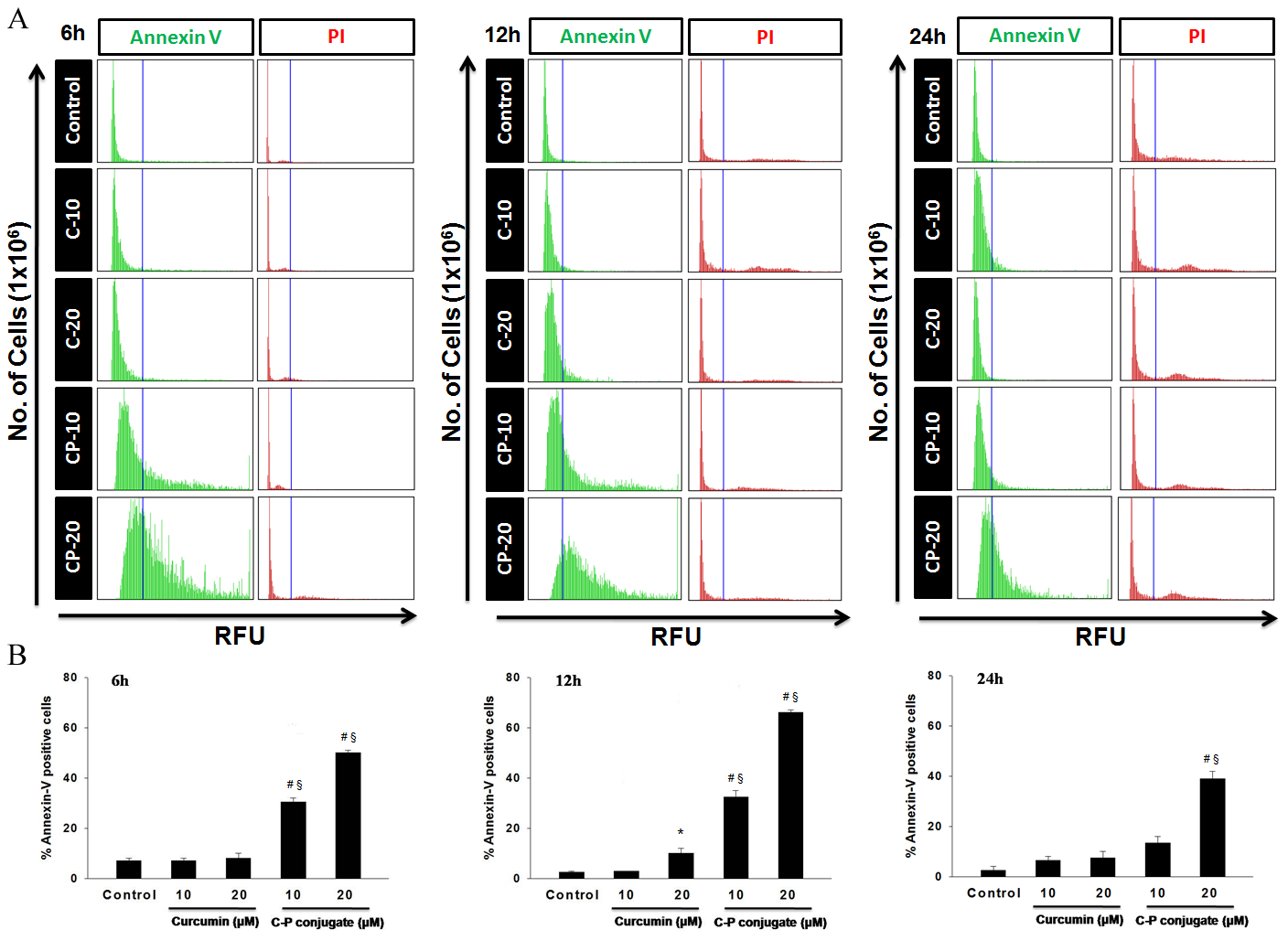

Supplement: S4 Fig — The HCT 116 cells were treated with 10 μM and 20 μM of curcumin and curcumin-PLGA conjugate for 6 h, 12 h and 24 h. Similarly, 2 μl DMSO was used as a vehicle control. Thereafter, apoptotic cell death was validated by using Annexin V-Alexa Fluor 488/ Propidium Iodide (PI) apoptosis detection kit, under automated image-based cytometer (Tali, Life Technologies, USA) according to manufacturer’s instructions. The cells were observed from 20 random fields for validation. (A) Representative images of Annexin-V/Propidium Iodide (PI) stained cells are shown in different panels. (B) The bar graphs represent percentage of Annexin-V and Propidium Iodide stained cells for late apoptosis. Error bars represent mean ±SEM of three independent experiments. Significant difference indicated as *p≤0.05 between untreated and curcumin treated cells; #p≤0.05 between untreated and curcumin-PLGA conjugate treated cells; §p≤0.05 between curcumin and curcumin-PLGA conjugate treated cells (One way ANOVA followed by Student Newman-Keuls multiple comparisons test). (TIF) [file pone.0117526.s004.tif]
